# Availability of critical care resources to treat patients with severe sepsis or septic shock in Africa: a self-reported, continent-wide survey of anaesthesia providers

**DOI:** 10.1186/cc9410

**Published:** 2011-01-10

**Authors:** Inipavudu Baelani, Stefan Jochberger, Thomas Laimer, Dave Otieno, Jane Kabutu, Iain Wilson, Tim Baker, Martin W Dünser

**Affiliations:** 1 Department of Anaesthesia and Critical Care Medicine, DOCS Hospital, Goma, Democratic Republic of Congo, Africa; 2 Klinik für Anästhesiologie der Technischen Universität München, Klinikum rechts der Isar, Ismaninger Strasse 22, 86175 München, Germany; 3 Medical University Vienna, Spitalgasse 23, 1090 Vienna, Austria; 4 Department of Anesthesiology and Critical Care Medicine, Kenyatta National Hospital, Hospital Road, 00202 Nairobi, Kenya, Africa; 5 Department of Anesthesiology, The Nairobi Hospital, Argwings Kodhek Road, 00100 Nairobi, Kenya, Africa; 6 Royal Devon and Exeter NHS Foundation Trust, Barrack Road, Exeter, EX2 5DW, UK; 7 Department of Physiology and Pharmacology, Karolinska Institute, Section for Anaesthesia and Intensive Care, Karolinska University Hospital, Huddinge, 14186 Stockholm, Sweden; 8 Department of Anaesthesiology, Perioperative and Intensive Care Medicine, Salzburg General Hospital and Paracelsus Private Medical University, Müllner Hauptstrasse 48, 5020 Salzburg, Austria

## Abstract

**Introduction:**

It is unknown whether resources necessary to implement the Surviving Sepsis Campaign guidelines and sepsis bundles are available in Africa. This self-reported, continent-wide survey compared the availability of these resources between African and high-income countries, and between two African regions (Sub-Sahara Africa *vs*. South Africa, Mauritius and the Northern African countries).

**Methods:**

The study was conducted as an anonymous questionnaire-based, cross-sectional survey among anaesthesia providers attending a transcontinental congress. Based on the respondents' country of practice, returned questionnaires were grouped into African and high-income countries. The questionnaire contained 74 items and evaluated all material resources required to implement the most recent Surviving Sepsis Campaign guidelines. Group comparisons were performed with the Chi^2^, Fisher's Exact or Mann Whitney *U *test, as appropriate.

**Results:**

The overall response rate was 74.3% (318/428). Three-hundred-seven questionnaires were analysed (African countries, *n *= 263; high-income countries, *n *= 44). Respondents from African hospitals were less likely to have an emergency room (85.5 *vs*. 97.7%, *P *= 0.03) or intensive care unit (73.8 *vs*. 100%, *P *< 0.001) than respondents from high-income countries. Drugs, equipment, and disposable materials required to implement the Surviving Sepsis Campaign guidelines or sepsis bundles were less frequently available in African than high-income countries. Of all African and Sub-Saharan African countries, 1.5% (4/263) and 1.2% (3/248) of respondents had the resources available to implement the Surviving Sepsis Campaign guidelines in entirety. The percentage of implementable recommendations was lower in African than in high-income countries (72.6 (57.7 to 87.7)% *vs*. 100 (100 to 100)%, *P *< 0.001) and lower in Sub-Saharan African countries than South Africa, Mauritius, and the Northern African countries (72.6 (56.2 to 86.3)% *vs*. 90.4 (71.2 to 94.5)%, *P *= 0.02).

**Conclusions:**

The results of this self-reported survey strongly suggest that the most recent Surviving Sepsis guidelines cannot be implemented in Africa, particularly not in Sub-Saharan Africa, due to a shortage of required hospital facilities, equipment, drugs and disposable materials. However, availability of resources to implement the majority of strong Surviving Sepsis Campaign recommendations and the sepsis bundles may allow modification of current sepsis guidelines based on available resources and implementation of a substantial number of life-saving interventions into sepsis care in Africa.

## Introduction

The annual incidence of sepsis is 750,000 cases in the United States and is increasing by 9% each year [1]. Sepsis is a major burden on the US healthcare system resulting in annual costs of $16.7 billion [2]. In Germany, an annual sepsis case load of 76 to 110 per 100,000 inhabitants has been estimated and held responsible for approximately 60,000 deaths per year [3]. Despite these startling figures from high-income countries, the largest part of the global burden of sepsis still occurs unrecognized by the Western medical community. Given that approximately 80% of the world's population live in low- or middle-income countries [4], it can be assumed that most sepsis cases occur outside the more economically developed world. While few reports on the outcome of sepsis in these countries exist, low hygienic standards, widespread malnutrition and a high incidence of bacterial, parasitic and HIV infection suggest a disproportionally high morbidity and mortality from sepsis in low- and middle-income countries [5]. Indeed, the latest global burden of disease report of the World Health Organization found that three infectious diseases (lower respiratory tract infection, diarrhoeal diseases, HIV/AIDS) range among the four most frequent causes of death in low-income countries [6].

During recent years, sepsis care in high-income countries has substantially improved due to extensive research efforts allowing novel insights into the pathophysiology and treatment of sepsis [7]. Current scientific evidence to improve the care of severe sepsis or septic shock patients is summarized in the Surviving Sepsis Campaign (SSC) guidelines [8,9], which are considered the gold standard of care in many countries. As repeatedly shown [10,11], implementation of the SSC guidelines into routine care can improve outcome from severe sepsis and septic shock. However, the possibility to implement the SSC guidelines in low- and middle-income countries has been questioned [5,12,13].

The aim of this survey was to compare availability of resources required to implement the SSC guidelines and sepsis bundles between anaesthesia providers from African and high-income countries as well as between anaesthesia providers from two African regions (Sub-Sahara Africa *vs*. South Africa, Mauritius and the Northern African countries). Based on personal experience and recent data, we hypothesized that the SSC guidelines could not effectively be implemented by African anaesthesia providers due to a lack of necessary hospital facilities, equipment, drugs and disposable materials.

## Materials and methods

This study was conducted as a self-reported, questionnaire-based, cross-sectional survey among anaesthesia providers attending the 4^th ^All Africa Anaesthesia Congress held in Nairobi/Kenya from 12 to 16 September 2009. During the opening plenary session of the congress, all attendants were informed about the purpose and anonymous design of the study together with the fact that its results would be published in a scientific journal. Considering that participation in the survey and information disclosure were voluntary, the study protocol did not undergo review by an Ethics Committee.

### Participants

On the first two days of the congress, questionnaires were haphazardly distributed to anaesthesia providers (both physicians and non-physicians) attending the congress. No incentives to complete the survey were offered. Throughout the congress, seven investigators were on site to collect the completed questionnaire responses and to be available for assistance in completing the form. Questionnaires retrieved from respondents practicing in an African (Table S1 in Additional file 1) or high-income country were eligible for study inclusion. High-income countries were defined according to the latest World Bank report [4]. Questionnaires completed by non-healthcare providers or those from non-African low- or middle-income countries were excluded.

## Survey instrument and data collection

The questionnaire was designed by all investigators based on the latest SSC guidelines. It was anonymous, contained 74 items grouped into seven main categories (general information, hospital facilities, drugs, patient monitoring, laboratory, equipment, disposables) and can be downloaded from the Additional file 1. Responses were classified as 'yes', 'no', 'don't know' for the category 'hospital facilities', and 'always', 'sometimes', 'never', 'don't know' for the remaining categories. The 'general information' category optionally required two open-ended text responses ('other hospital type' and 'other medical grade') which were retrospectively coded by two study investigators. The study questionnaire was written in English, underwent pre-testing by the investigators and subsequently pilot testing by 10 anaesthesia providers in two African countries (Hospital of Kisumu/Kenya, *n *= 5; Muhimbili Hospital, Dar-Es-Salaam/Tanzania, *n *= 5). For the pilot test, anaesthesia providers were asked to complete and examine the questionnaire with regard to its flow, salience, acceptability and administrative ease. Inter-rater reliability was assessed for all five respondents from each hospital and yielded a Cohen's Kappa of 0.71. Based on the results of the pilot testing and individual feedback, the questionnaire was modified. Finally, it was again reviewed and approved by all investigators.

### Study variables

The main study variable was availability of resources necessary to implement the latest SSC guidelines and their sepsis resuscitation/management bundles [9]. Prior to the survey, hospital facilities, equipment, drugs and disposable materials required to implement individual SSC recommendations and sepsis bundles were defined by consensus of the investigators (Table S2 in Additional file 1). In order to consistently implement the SSC guidelines, resources had to be 'always' available. Resources 'sometimes' or 'never' available, as well as those respondents who did not know whether they were available at their hospital were considered insufficient to implement the SSC guidelines. Furthermore, the percentage of implementable recommendations of the SSC guidelines was calculated for each returned questionnaire.

### Study cohorts and survey goals

Based on the respondents' countries of practice, questionnaires were grouped into African and high-income countries. Furthermore, African countries were sub-grouped into two regions: 1) Sub-Saharan African countries generally representing low-income countries, and 2) South Africa, Mauritius, and the Northern African countries rated as middle-income countries according to the World Bank [4]. With few exceptions, this economy-based country classification by the World Bank correlates well with the quality and development of the national health care systems [4].

The primary goal of our survey was to compare the availability of each resource to implement the SSC guidelines, the percentage of implementable guidelines, and the possibility to implement the SSC guidelines (Grade 1 and 2 recommendations) and their associated sepsis bundles (resuscitation and management bundles) between respondents and hospitals from African and high-income countries. Comparison of the same variables between respondents from Sub-Saharan African countries and South Africa, Mauritius, and the Northern African countries was considered the secondary survey goal.

### Data processing and statistical analysis

Questionnaires were manually entered into a centralized database. After random cross-checking, the database was re-checked by calculating minimum and maximum values of each question in order to detect entry errors.

The SPSS software package (SPSS 13.0.1; SPSS Inc., Chicago, IL, USA) was used for statistical analysis. Frequencies based on the number of completed questions (some questions were not completed by all respondents) were calculated for all categorical data. Continuous variables are presented as median values with interquartile ranges. Categorical and non-continuous variables were compared between groups using the Chi^2^- or Fisher's Exact test, as appropriate. For comparisons of resource availability, only 'always', 'sometimes' and 'never' choices were statistically evaluated. Group comparisons of continuous data were performed with the Mann Whitney *U *test. *P*-values < 0.05 were considered to indicate statistical significance.

## Results

**Figure 1:**
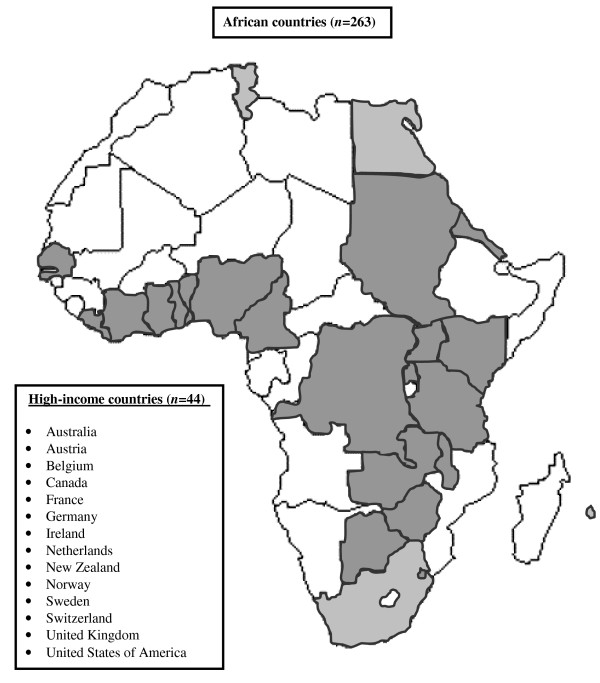
**Countries of practice of survey participants**. Sub-Saharan African countries (*n *= 248) are marked in dark grey. South Africa, Mauritius and the Northern African countries (*n *= 15) are marked in light grey.

Questionnaires were randomly distributed to 428 of 832 congress attendants. A total of 318 questionnaires were returned (overall response rate, 74.3%). Eleven questionnaires had to be excluded because respondents practicing in non-African middle-income countries (India, *n *= 5; Romania, *n *= 2), were returned blank (*n *= 3), or were completed by non-healthcare providers (*n *= 1). Finally, 307 questionnaires were statistically analysed (African countries, *n *= 263; high-income countries, *n *= 44). Respondents from 185 hospitals located in 14 high-income and 24 African countries (45.3% of all 53 African countries) were included (Sub-Saharan African countries, *n *= 248; South Africa, Mauritius, and the Northern African countries, *n *= 15) (Figure 1). The median (interquartile range) number of respondents per hospital, respondents per country, and respondents' hospitals per country was 1 (1 to 1), 2 (1 to 5), and 2 (1 to 4), respectively. One hundred-nine questionnaires (35.5%) were partially incomplete. The median number of missing responses per incomplete questionnaire was 1 (interquartile range, 1 to 3).

Characteristics of respondents and their hospitals are summarized in Table 1. Significant differences between respondents from African and high-income countries were observed in regards to the respondent's specialty, hospital type and size, as well as the availability of an emergency room and an intensive care unit. Differences in the availability of an intensive care unit were observed between Sub-Saharan African countries and South Africa, Mauritius, and the Northern African countries (Table S3 in Additional file 1).

**Table 1:** Characteristics of respondents and their hospitals

		African countries	High-income countries	*P*-value
***n***		263	44	
**Specialty of respondent**	*n (%)*			0.002*
*Physician anaesthetist*		150 (57)	35 (81.8)	
*Non-physician anaesthetist*		92 (35)	5 (11.4)	
*Other physician*		6 (2.3)	3 (6.8)	
*Other*		15 (5.7)	0	
**Type of hospital**	*n (%)*			0.01*
*University teaching*		117 (44.5)	19 (43.2)	
*Regional/Provincial*		30 (11.4)	11 (25)	
*District*		34 (12.9)	9 (20.5)	
*Private*		61 (23.2)	3 (6.8)	
*Other*		21 (8)	2 (4.6)	
**Size of hospital**	*(beds)*	350 (200 to 1,000)	600 (388 to 800)	0.03*
**Availability of hospital facilities**	*n (%)*			
*Emergency room*		225 (85.6)	43 (97.7)	0.03*
*Operation theatre*		260 (98.9)	44 (100)	1
*Intensive care unit*		194 (73.8)	44 (100)	<0.001*

*, significant group difference

Data are given as median values with interquartile ranges, if not otherwise indicated.

Comparisons were made with a Fisher's Exact or a Mann Whitney U test (size of hospital).

Respondents from African countries reported to have drugs (Table 2), equipment (Table 3), and disposable materials (Table 4) required to implement the SSC guidelines less frequently available than respondents from high-income countries. Certain drugs, equipment, and disposable materials (Table S4-6 in Additional file 1) were less frequently available for respondents from Sub-Saharan African countries compared to those from South Africa, Mauritius, and the Northern African countries. The possibility to perform thick drop analysis to diagnose malaria was the single resource more frequently available for respondents from African countries compared to high-income countries and for respondents from Sub-Saharan African countries compared to South Africa, Mauritius, and the Northern African countries.

**Table 2:** Availability of drugs to implement the surviving sepsis campaign guidelines

	African countries *N *= 263				High-income countries *N *= 44				*P*-value
	*Always*	*Some-times*	*Never*	*Don't know*	*Always*	*Some-times*	*Never*	*Don't know*	
**Oxygen**	243 (93.8)	15 (5.8)	1 (0.4)	0	44 (100)	0	0	0	0.24
**Antibiotics**									
*Ampicilline*	201 (78.5)	47 (18.4)	5 (2)	3 (1.2)	44 (100)	0	0	0	0.004*
*Gentamycine*	243 (92.4)	19 (7.2)	0	1 (0.4)	44 (100)	0	0	0	0.07
*3*^*rd*^*/4*^*th *^*Gen Cephalosporine*	203 (77.5)	57 (21.8)	1 (0.4)	1 (0.4)	44 (100)	0	0	0	0.002*
*Piperacilline*	65 (26.4)	40 (16.3)	83 (33.7)	58 (23.6)	43 (97.7)	0	0	1 (2.3)	<0.001*
*Carbapenem*	79 (32.1)	46 (18.7)	68 (27.6)	53 (21.5)	43 (97.7)	0	0	1 (2.3)	<0.001*
**IV fluids**									
*Crystalloids*	254 (96.6)	9 (3.4)	0	0	44 (100)	0	0	0	0.21
*Colloids*	158 (60.8)	81 (31.2)	16 (6.2)	5 (1.9)	44 (100)	0	0	0	<0.001*
**Blood products**									
*Red blood cells*	206 (79.5)	51 (19.7)	1 (0.4)	1 (0.4)	44 (100)	0	0	0	0.005*
*Fresh frozen plasma*	109 (42.6)	81 (31.6)	59 (23)	7 (2.7)	44 (100)	0	0	0	<0.001*
*Platelets*	69 (27.2)	93 (36.6)	79 (31.1)	13 (5.1)	44 (100)	0	0	0	<0.001*
**Cardiovascular drugs**									
*Noradrenaline*	119 (46.7)	60 (23.5)	67 (26.3)	9 (3.5)	44 (100)	0	0	0	<0.001*
*Dopamine*	133 (51.4)	68 (26.3)	52 (20.1)	6 (2.3)	44 (100)	0	0	0	<0.001*
*Dobutamine*	94 (37.2)	67 (26.5)	77 (30.4)	15 (5.9)	44 (100)	0	0	0	<0.001*
*Adrenaline*	255 (97.3)	7 (2.7)	0	0	44 (100)	0	0	0	0.27
*Hydrocortisone*	252 (96.2)	9 (3.4)	0	1 (0.4)	44 (100)	0	0	0	0.21
*Vasopressin*	76 (29.8)	66 (25.9)	93 (36.5)	20 (7.8)	43 (97.7)	0	0	1 (2.3)	<0.001*
**Anesthetic/sedative drugs**									
*Thiopentone*	243 (93.1)	11 (4.2)	7 (2.7)	0	43 (97.7)	1 (2.3)	0	0	0.44
*Propofol*	154 (59)	71 (27.2)	36 (13.8)	0	43 (97.7)	1 (2.3)	0	0	<0.001*
*Succinylcholine*	242 (93.4)	9 (3.5)	7 (2.7)	1 (0.4)	44 (100)	0	0	0	0.24
*ND Muscle Relaxant*	185 (70.6)	59 (22.5)	18 (6.9)	0	44 (100)	0	0	0	<0.001*
*IV opiate/opioide*	208 (79.7)	50 (19.2)	3 (1.1)	0	44 (100)	0	0	0	0.004*
*Diazepam*	251 (95.8)	11 (4.2)	0	0	44 (100)	0	0	0	0.17
*Midazolam*	138 (52.9)	72 (27.6)	48 (18.4)	3 (1.1)	44 (100)	0	0	0	<0.001*
**Others**									
*Insulin*	232 (89.2)	27 (10.4)	1 (0.4)	0	44 (100)	0	0	0	0.07
*Heparin or LMWH*	174 (67.7)	56 (21.8)	21 (8.2)	6 (2.3)	44 (100)	0	0	0	<0.001*
*H2-Blockers*	182 (70.3)	64 (24.7)	9 (3.5)	4 (1.5)	44 (100)	0	0	0	<0.001*
*Proton pump inhibitor*	145 (55.1)	82 (31.2)	18 (7.1)	10 (3.9)	42 (95.5)	0	0	2 (4.5)	<0.001*
*Activated protein C*	15 (6.1)	32 (13)	139 (56.3)	61 (24.7)	39 (88.6)	1 (2.3)	2 (4.5)	2 (4.5)	<0.001*

*, significant group difference ('don't know' choices were not included in statistical comparisons); H2, histamine receptor 2; IV, intravenous; LMWH, low molecular weight heparin; ND, non-depolarizing.

All data are given as absolute values and percentages of completed responses.

**Table 3:** Availability of equipment to implement the surviving sepsis campaign guidelines

	African countries *N *= 263				High-income countries *N *= 44				*P*-value
	*Always*	*Some-times*	*Never*	*Don't know*	*Always*	*Some-times*	*Never*	*Don't know*	
**Diagnostic equipment**									
*X-ray*	236 (90.8)	14 (5.4)	10 (3.8)	0	44 (100)	0	0	0	0.11
*Sonography*	216 (83.4)	17 (6.6)	25 (9.7)	1 (0.4)	43 (97.7)	1 (2.3)	0	0	0.04*
*Echocardiography*	152 (60.6)	30 (12)	61 (24.3)	8 (3.2)	40 (90.9)	4 (9.1)	0	0	<0.001*
**Laboratory investigations**									
*Thick drop*	251 (96.5)	4 (1.5)	3 (1.2)	2 (0.8)	35 (79.5)	3 (6.8)	5 (11.4)	1 (2.3)	<0.001*
*Gram stain*	238 (92.2)	18 (7)	2 (0.8)	0	43 (97.7)	1 (2.3)	0	0	0.41
*Microbiological cultures*	193 (75.1)	44 (17.1)	19 (7.4)	1 (0.4)	44 (100)	0	0	0	0.001*
*Antibiogram*	181 (70.4)	51 (19.8)	21 (8.2)	4 (1.6)	44 (100)	0	0	0	<0.001*
*Blood sugar*	242 (93.1)	17 (6.5)	1 (0.4)	0	44 (100)	0	0	0	0.2
*Blood gas analysis*	110 (43.5)	48 (19)	89 (35.2)	6 (2.4)	44 (100)	0	0	0	<0.001*
*Lactate*	64 (25.7)	60 (24.1)	92 (36.9)	33 (13.3)	43 (97.7)	1 (2.3)	0	0	<0.001*
*Blood count*	227 (87.6)	27 (10.4)	4 (1.5)	1 (0.4)	44 (100)	0	0	0	0.05
*Creatinine*	201 (78.2)	40 (15.6)	15 (5.8)	1 (0.4)	44 (100)	0	0	0	0.003*
*Bilirubin*	194 (74.6)	46 (17.7)	18 (6.9)	2 (0.8)	44 (100)	0	0	0	0.001*
*Prothrombin time/INR*	176 (68.5)	39 (15.2)	36 (14)	6 (2.3)	44 (100)	0	0	0	<0.001*
*Other coagulation tests*	149 (60.1)	59 (23.8)	29 (11.7)	11 (4.4)	44 (100)	0	0	0	<0.001*
**Monitoring equipment**									
*Body temperature*	205 (79.2)	50 (19.3)	2 (0.8)	2 (0.8)	42 (95.5)	2 (4.5)	0	0	0.04*
*Non-invasive blood pressure*	241 (93.8)	11 (4.3)	5 (1.9)	0	43 (97.7)	1 (2.3)	0	0	0.52
*Invasive blood pressure*	58 (23.1)	84 (33.5)	102 (40.6)	7 (2.8)	41 (93.2)	3 (6.8)	0	0	<0.001*
*Oxygen saturation*	199 (76.8)	42 (16.2)	18 (6.9)	0	44 (100)	0	0	0	<0.001*
*Central venous pressure*	87 (33.9)	78 (30.4)	90 (35)	2 (0.8)	41 (93.2)	3 (6.8)	0	0	<0.001*
*Cardiac output*	30 (12)	62 (24.8)	146 (58.4)	12 (4.8)	37 (84.1)	5 (11.4)	2 (4.5)	0	<0.001*
*Pulmonary arterial pressure*	22 (8.6)	45 (17.6)	174 (68)	15 (5.9)	34 (77.3)	8 (18.2)	2 (4.5)	0	<0.001*
*Endtidal carbon dioxyde*	99 (38.2)	78 (30.1)	78 (30.1)	4 (1.5)	43 (97.7)	1 (2.3)	0	0	<0.001*
**Other equipment**									
*Mechanical ventilator*	184 (71.9)	38 (14.8)	34 (13.3)	34 (13.3)	44 (100)	0	0	0	<0.001*
*Syringe pump*	138 (53.9)	53 (20.7)	61 (23.8)	4 (1.6)	44 (100)	0	0	0	<0.001*
*Fluid infuser*	126 (49)	61 (23.7)	67 (26.1)	3 (1.2)	44 (100)	0	0	0	<0.001*
*Peritoneal dialysis*	91 (36)	50 (19.8)	98 (38.7)	14 (5.5)	38 (86.4)	3 (6.8)	1 (2.3)	2 (4.5)	<0.001*
*Hemodialysis/Hemofiltration*	111 (43.2)	26 (10.1)	110 (42.8)	10 (3.9)	43 (97.7)	1 (2.3)	0	0	<0.001*

*, significant group difference ('don't know' choices were not included in statistical comparisons); INR, international normalized ratio; All data are given as absolute values and percentages of completed responses.

**Table 4:** Availability of disposable material to implement the surviving sepsis campaign guidelines

	African countries *N *= 263				High-income countries *N *= 44				*P*-value
	*Always*	*Some-times*	*Never*	*Don't know*	*Always*	*Some-times*	*Never*	*Don't know*	
**Disposable material**									
*IV cannula*	253 (97.3)	6 (2.3)	1 (0.4)	0	44 (100)	0	0	0	0.55
*IV fluid giving set*	253 (97.7)	3 (1.2)	3 (1.2)	0	44 (100)	0	0	0	0.6
*Urinary catheter*	249 (95.4)	12 (4.6)	0	0	44 (100)	0	0	0	0.15
*Nasogastric tube*	246 (94.3)	15 (5.7)	0	0	44 (100)	0	0	0	0.1
*Endotracheal tube*	251 (96.5)	6 (2.3)	3 (1.2)	0	44 (100)	0	0	0	0.46
*Oxygen face mask*	252 (96.6)	7 (2.7)	2 (0.8)	0	44 (100)	0	0	0	0.46
*Oxygen nasal cannula*	212 (81.5)	37 (14.2)	9 (3.5)	2 (0.8)	44 (100)	0	0	0	0.01*
*Central venous catheter*	126 (48.8)	64 (24.8)	63 (24.4)	5 (1.9)	44 (100)	0	0	0	<0.001*
*Antithrombotic stockings*	65 (25.4)	64 (25)	94 (36.7)	33 (12.9)	43 (97.7)	1 (2.3)	0	0	<0.001*

*, significant group difference ('don't know' choices were not included in statistical comparisons); IV, intravenous.

Of all African respondents and hospitals, four (1.5%) and two (1.4%), respectively, stated to have the resources available to consistently implement the SSC guidelines or any of their sepsis resuscitation and management bundles (Tables 5 and 6). Respondents and hospitals from African countries less frequently had all resources available to implement the SSC guidelines (Grade 1 and 2 recommendations) than respondents from high-income countries. The percentage of implementable grade 1 and 2 recommendations was lower in respondents and hospitals from African compared to high-income countries (Tables 5 and 6). The percentage of implementable SSC guidelines was different between African respondents working in hospital of different types (<0.01 for all comparisons; data not shown). Resources to implement the SSC guidelines (grade 1 and 2 recommendations) tended to be less frequently available for respondents from Sub-Saharan African countries than those from South Africa, Mauritius, and the Northern African countries (Table 7).

**Table 5:** Possibility to implement the surviving sepsis campaign guidelines

		African countries	High-income countries	*P*-value
**Respondents**	*n*	263	44	
**Possibility to implement the SSC guidelines in entirety**	*n (%)*	4 (1.5)	36 (81.8)	<0.001*
*Percentage of implementable recommendations/suggestions*	*(%)*	72.6 (57.5 to 87.7)	100 (100 to 100)	<0.001*
**Possibility to implement all Grade 1 recommendations**	*n (%)*	15 (5.7)	40 (90.9)	<0.001*
*Percentage of implementable Grade 1 recommendations*	*(%)*	80.8 (63.5 to 88.5)	100 (100 to 100)	<0.001*
**Possibility to implement all Grade 1A and 1B recommendations**	*n (%)*	30 (11.4)	41 (93.2)	<0.001*
*Percentage of implementable Grade 1A and 1B recommendations*	*(%)*	87.5 (70.8 to 95.8)	100 (100 to 100)	<0.001*
**Possibility to implement all Grade 1C and 1D recommendations**	*n (%)*	26 (9.9)	40 (90.9)	<0.001*
*Percentage of implementable Grade 1C and 1D recommendations*	*(%)*	71.4 (57.1 to 89.3)	100 (100 to 100)	<0.001*
**Possibility to implement all Grade 2 recommendations**	*n (%)*	4 (1.5)	36 (81.8)	<0.001*
*Percentage of implementable Grade 2 recommendations*	*(%)*	57.1 (38.1 to 81)	100 (100 to 100)	<0.001*
**Possibility to implement all sepsis resuscitation bundles**	*n (%)*	43 (16.3)	41 (93.2)	<0.001*
*Bundle element "Lactate"*	*n (%)*	64 (24.3)	43 (97.7)	<0.001*
*Bundle element "Cultures"*	*n (%)*	188 (71.5)	44 (100)	<0.001*
*Bundle element "Antibiotics"*	*n (%)*	204 (77.6)	44 (100)	<0.001*
*Bundle element "Hypotension"*	*n (%)*	238 (90.5)	44 (100)	0.03*
*Bundle element "CVP/ScvO2"*	*n (%)*	70 (26.6)	41 (93.2)	<0.001*
**Possibility to implement all sepsis management bundles**	*n (%)*	12 (4.6)	39 (88.6)	<0.001*
*Bundle element "Steroids"*	*n (%)*	252 (95.8)	44 (100)	0.17
*Bundle element "rhAPC"*	*n (%)*	15 (5.7)	39 (88.6)	<0.001*
*Bundle element "Glucose"*	*n (%)*	221 (84)	44 (100)	0.004*
*Bundle element "Plateau Pressure"*	*n (%)*	182 (69.2)	44 (100)	<0.001*

SSC, Surviving Sepsis Campaign; *, significant group difference. Data are given as median values with interquartile ranges, if not otherwise indicated. The Surviving Sepsis Campaign categorized their recommendations using the GRADE system which classifies recommendations as strong (grade 1) or weak (grade 2). Furthermore, the system Classifies the quality of evidence as high (grade A), moderate (grade B), low (grade C), or very low (grade D).The grade of strong or weak is considered of greater clinical importance than a difference in letter level of quality of evidence. Sepsis Resuscitation Bundles: Element "Lactate", measurement of serum lactate; Element "Cultures", obtainment of blood cultures prior to antibiotic administration; Element "Antibiotics", administration of broad-spectrum antibiotics within three hours of emergency department admission and within one hour of non-emergency department admission; Element "Hypotension", treatment of hypo-tension and/or elevated lactate with fluids; Element "CVP/ScvO2", maintenance of adequate central venous pressure and central venous oxygen saturation. Sepsis Management Bundles: Element "Steroids", administration of low-dose steroids for septic shock in accordance with a standardized ICU policy; Element "rhAPC", administration of recombinant human activated protein C in accordance with a standardized ICU policy; Element "Glucose", maintenance of blood glucose control lower limit of normal, but <180 mg/dL (10 mmol/L); Element "Plateau Pressure", maintenance of a median inspiratory plateau pressure <30 cmH2O for mechanically ventilated patients.

**Table 6:** Possibility to implement the surviving sepsis campaign guidelines per hospital

		African countries	High-income countries	*P*-value
**Hospital**	*n*	143	42	
**Possibility to implement the ssc guidelines in entirety**	*n (%)*	2 (1.4)	34 (81)	<0.001*
*Percentage of implementable recommendations/suggestions*	*(%)*	67.1 (52.1 to 80.8)	100 (100 to 100)	<0.001*
**Possibility to implement all Grade 1 recommendations**	*n (%)*	5 (3.5)	38 (90.5)	<0.001*
*Percentage of implementable Grade 1 recommendations*	*(%)*	75 (59.6 to 84.6)	100 (100 to 100)	<0.001*
**Possibility to implement all Grade 1A and 1B recommendations**	*n (%)*	16 (11.2)	39 (92.9)	<0.001*
*Percentage of implementable Grade 1A and 1B recommendations*	*(%)*	83.3 (66.7 to 91.7)	100 (100 to 100)	<0.001*
**Possibility to implement all Grade 1C and 1D recommendations**	*n (%)*	9 (6.3)	38 (90.5)	<0.001*
*Percentage of implementable Grade 1C and 1D recommendations*	*(%)*	71.4 (57.1 to 82.1)	100 (100 to 100)	<0.001*
**Possibility to implement all Grade 2 recommendations**	*n (%)*	2 (1.4)	34 (81)	<0.001*
*Percentage of implementable Grade 2 recommendations*	*(%)*	47.6 (33.3 to 71.4)	100 (100 to 100)	<0.001*
**Possibility to implement all sepsis resuscitation bundles**	*n (%)*	14 (9.8)	39 (92.9)	<0.001*
*Bundle element "Lactate"*	*n (%)*	31 (21.7)	41 (97.6)	<0.001*
*Bundle element "Cultures"*	*n (%)*	97 (67.8)	42 (100)	<0.001*
*Bundle element "Antibiotics"*	*n (%)*	36 (25.2)	42 (100)	<0.001*
*Bundle element "Hypotension"*	*n (%)*	129 (90.2)	42 (100)	0.04*
*Bundle element "CVP/ScvO2"*	*n (%)*	28 (19.6)	39 (92.9)	<0.001*
**Possibility to implement all sepsis management bundles**	*n (%)*	3 (2.1)	37 (88.9)	<0.001*
*Bundle element "Steroids"*	*n (%)*	137 (95.8)	42 (100)	0.18
*Bundle element "rhAPC"*	*n (%)*	5 (3.5)	37 (88.9)	<0.001*
*Bundle element "Glucose"*	*n (%)*	115 (80.4)	42 (100)	0.002*
*Bundle element "Plateau Pressure"*	*n (%)*	85 (59.4)	42 (100)	<0.001*

SSC, Surviving Sepsis Campaign; *, significant group difference. Data are given as median values with interquartile ranges, if not otherwise indicated. The Surviving Sepsis Campaign categorized their recommendations using the GRADE system which classifies recommendations as strong (grade 1) or weak (grade 2). Furthermore, the system Classifies the quality of evidence as high (grade A), moderate (grade B), low (grade C), or very low (grade D).The grade of strong or weak is considered of greater clinical importance than a difference in letter level of quality of evidence. Sepsis Resuscitation Bundles: Element "Lactate", measurement of serum lactate; Element "Cultures", obtainment of blood cultures prior to antibiotic administration; Element "Antibiotics", administration of broad-spectrum antibiotics within three hours of emergency department admission and within one hour of non-emergency department admission; Element "Hypotension", treatment of hypo-tension and/or elevated lactate with fluids; Element "CVP/ScvO2", maintenance of adequate central venous pressure and central venous oxygen saturation. Sepsis Management Bundles: Element "Steroids", administration of low-dose steroids for septic shock in accordance with a standardized ICU policy; Element "rhAPC", administration of recombinant human activated protein C in accordance with a standardized ICU policy; Element "Glucose", maintenance of blood glucose control lower limit of normal, but <180 mg/dL (10 mmol/L); Element "Plateau Pressure", maintenance of a median inspiratory plateau pressure <30 cmH2O for mechanically ventilated patients.

**Table 7:** Possibility to implement the surviving sepsis campaign guidelines in African countries

		Sub-Saharan African countries	South Africa/Mauritius/Northern African countries	*P*-value
**Respondents**	*n*	248	15	
**Possibility to implement the SSC guidelines in entirety**	*n (%)*	3 (1.2)	1 (6.7)	0.09
*Percentage of implementable recommendations/suggestions*	*(%)*	72.6 (56.2 to 86.3)	90.4 (71.2 to 94.5	0.02*
**Possibility to implement all Grade 1 recommendations**	*n (%)*	12 (4.8)	3 (20)	0.01*
*Percentage of implementable Grade 1 recommendations*	*(%)*	78.9 (63.5 to 88.5)	94.2 (76.9 to 98.1)	0.03*
**Possibility to implement all Grade 1A and 1B recommendations**	*n (%)*	27 (10.9)	3 (20)	0.28
*Percentage of implementable Grade 1A and 1B recommendations*	*(%)*	87.5 (70.8 to 95.8)	95.8 (79.2 to 95.8)	0.09
**Possibility to implement all Grade 1C and 1D recommendations**	*n (%)*	20 (8.1)	6 (40)	<0.001*
*Percentage of implementable Grade 1C and 1D recommendations*	*(%)*	71.4 (57.1 to 85.7)	92.9 (67.9 to 100)	0.02*
**Possibility to implement all Grade 2 recommendations**	*n (%)*	3 (1.2)	1 (6.7)	0.09
*Percentage of implementable Grade 2 recommendations*	*(%)*	52.4 (38.1 to 76.2)	81 (66.7 to 90.5)	0.009*
**Possibility to implement all sepsis resuscitation bundles**	*n (%)*	36 (14.5)	7 (46.7)	0.001*
*Bundle element "Lactate"*	*n (%)*	57 (23)	7 (46.7)	0.04*
*Bundle element "Cultures"*	*n (%)*	176 (71)	12 (80)	0.45
*Bundle element "Antibiotics"*	*n (%)*	189 (76.2)	15 (100)	0.03*
*Bundle element "Hypotension"*	*n (%)*	225 (90.7)	13 (86.7)	0.6
*Bundle element "CVP/ScvO2"*	*n (%)*	60 (24.2)	10 (66.7)	<0.001*
**Possibility to implement all sepsis management bundles**	*n (%)*	11 (4.4)	1 (6.7)	0.69
*Bundle element "Steroids"*	*n (%)*	237 (95.6)	15 (100)	0.41
*Bundle element "rhAPC"*	*n (%)*	13 (5.2)	2 (13.3)	0.19
*Bundle element "Glucose"*	*n (%)*	208 (83.9)	13 (86.7)	0.77
*Bundle element "Plateau Pressure"*	*n (%)*	172 (69.4)	10 (66.7)	0.83

SSC, Surviving Sepsis Campaign; *, significant group difference. Data are given as median values with interquartile ranges, if not otherwise indicated. The Surviving Sepsis Campaign categorized their recommendations using the GRADE system which classifies recommendations as strong (grade 1) or weak (grade 2). Furthermore, the system Classifies the quality of evidence as high (grade A), moderate (grade B), low (grade C), or very low (grade D).The grade of strong or weak is considered of greater clinical importance than a difference in letter level of quality of evidence. Sepsis Resuscitation Bundles: Element "Lactate", measurement of serum lactate; Element "Cultures", obtainment of blood cultures prior to antibiotic administration; Element "Antibiotics", administration of broad-spectrum antibiotics within three hours of emergency department admission and within one hour of non-emergency department admission; Element "Hypotension", treatment of hypotension and/or elevated lactate with fluids; Element "CVP/ScvO2", maintenance of adequate central venous pressure and central venous oxygen saturation. Sepsis Management Bundles: Element "Steroids", administration of low-dose steroids for septic shock in accordance with a standardized ICU policy; Element "rhAPC", administration of recombinant human activated protein C in accordance with a standardized ICU policy; Element "Glucose", maintenance of blood glucose control lower limit of normal, but <180 mg/dL (10 mmol/L); Element "Plateau Pressure", maintenance of a median inspiratory plateau pressure <30 cmH2O for mechanically ventilated patients.

## Discussion

The results of this continent-wide, cross-sectional, self-reported, questionnaire-based survey indicate that while almost all respondents from high-income countries reported to be able to implement the latest SSC guidelines, only a small percentage of African respondents stated to have the required facilities, equipment, drugs and disposable materials consistently available to implement the SSC in entirety. These results remained unchanged when comparisons were made between hospitals instead of respondents. Supporting our hypothesis, these data imply that the SSC guidelines cannot be implemented in entirety by African, particularly not by Sub-Saharan African, respondents due to a lack of necessary resources. However, with a wide variability among African respondents, resources appear to be available to implement approximately three-quarter of individual SSC recommendations. These results are important for the future management of sepsis care in Africa.

The finding that only 1.5% of African respondents and even less from Sub-Sahara Africa (1.2%) reported to have the resources constantly available to treat sepsis patients according to the latest SSC guidelines in entirety is striking on the first sight. However, almost three-quarters of individual SSC recommendations and sepsis resuscitation/management bundles could be implemented by African respondents. The interquartile range of implementable guidelines (57.5 to 87.7%) was wide, suggesting considerable heterogeneity among respondents. When interpreting these figures, it needs to be taken into account that 16 of the 73 (21.9%) recommendations of the SSC guidelines are either passive ('do not use') or require no resources at all. These results underline earlier criticism that SSC guidelines may not be feasible in low- or middle-income countries [5,12,13]. Our finding that not even all respondents of high-income countries reported to have resources available to implement the SSC guidelines was unexpected. However, this was due to the lack of selected resources (for example, vasopressin or activated protein C). The median percentage of implementable SSC recommendations reported by respondents from high-income countries was 100%.

Lack of resources required to implement the SSC guidelines and sepsis bundles by African anaesthesia providers was not confined to specific materials but appears multi-faceted. For example, 25% of respondents stated that their hospital did not have an intensive care unit. Similarly, an emergency room, a key hospital facility to timely recognize, triage and treat sepsis patients [14,15], was not available in the hospitals of 15% of respondents. Nearly all materials necessary to implement the SSC guidelines were less frequently available for African respondents compared to those from high-income countries. Particularly shocking is the inconsistent supply or lack of basic resources such as oxygen, fluids and broad-spectrum antibiotics or essential disposables and monitoring equipment reported by some respondents. As suggested before [16,17], the lack of resources was especially pronounced for respondents practicing in Sub-Sahara Africa.

Although our survey did not specifically ask whether respondents routinely care for sepsis patients, several earlier studies reported that anaesthetists are crucially involved in intensive care medicine and in the care of critically ill sepsis patients in Africa [16,18-22]. Nonetheless, it is notable that a relevant number of respondents stated that they did not know whether certain resources to treat sepsis were available at their hospital. Given the drastic shortage of intensive care capacities in some African countries such as in Zambia [16], it must be assumed that many clinicians from other medical specialties routinely care for critically ill sepsis patients, for example, on normal hospital wards. Considering this, our survey only reflects the possibility of African anaesthesia providers to implement the SSC guidelines and cannot yield data on resource availability of other African health care workers to care for sepsis patients. Furthermore, the study specifically assessed the availability of resources required to implement the SSC guidelines and did not appraise the process of clinical sepsis care itself. It remains unknown whether the scarce resources are used appropriately. Similarly, our survey only evaluated the qualitative availability of resources. Although some resources may be available their quantity could still not be adequate to treat all patients with sepsis.

The wide-ranging lack of resources as reflected by our study entails that several potentially life-saving interventions cannot be applied to sepsis patients in Africa. Although no causative relationship can be drawn, high sepsis mortality as reported from low- or middle-income countries may be due to a lack of hospital facilities, equipment, drugs and disposable materials. Accordingly, a Tunisian study including 100 septic shock patients reported a lethality of 82% [23]. Similarly, mortality from severe sepsis was 80 and 92% in a tertiary centre in Pakistan [24] and Turkey [25], respectively. Cheng *et al*. observed a mortality of 90% in patients with severe sepsis due to melioidosis in a Thai provincial hospital [26].

Our survey has important limitations. First, although the questionnaire used in this survey underwent pilot testing, no assessment of test-retest reliability was performed. Together with the lack of clinical sensibility testing of the questionnaire, this limits the validity of the results [27]. Second, by including only anaesthesia providers attending a continent-wide congress our survey most likely experienced a relevant selection bias due to respondent clustering. Accordingly, the proportion of African respondents working either at university or private hospitals was high, while barely 25% came from provincial or district hospitals which make up the crucial part of healthcare services in Africa. Some rural African hospitals do not even have anaesthesia providers available [28]. Therefore, our results almost certainly overestimate the true situation of resource availability to treat sepsis in Africa, particularly in rural hospitals. Recent studies from Uganda [17] and Zambia [16] reported a much lower availability of basic monitoring devices or equipment than observed in our survey. Third, the present survey only evaluated the availability of material resources and not healthcare workers. Shortage of sufficiently trained healthcare providers is another threat to adequate patient care in Africa [28-30]. Even in high-income countries, barriers to implementation of the SSC guidelines may be related to inadequate staffing [31]. Fourth, although our survey included respondents from half of African countries, its results must not be extrapolated to all of Africa. Additionally, the questionnaire did not assess the availability of resources necessary to manage children with sepsis. Since special-sized disposable materials and equipment are required, it is likely that resources necessary to care for critically ill children with sepsis are even less frequently available. Finally, we cannot exclude that some respondents may have misunderstood certain questions despite the availability of 'don't know' choices. Although the congress was held in English, it is possible that language problems contributed to misunderstandings. Some respondents chose to answer only questions they could respond with 'yes' or 'always' leaving the remaining questions blank.

Several possibilities exist to improve sepsis care in Africa. While a consistent supply of resources to implement the SSC guidelines in its entirety would not only be logistically and economically unrealistic but also require training of health care providers to use so far unavailable resources, modification of existing sepsis guidelines could help see that currently available resources are used according to the latest clinical evidence. This may be particularly relevant for therapeutic interventions with a high chance of improving patient survival. Optimistically, respondents reported to have resources constantly available to implement up to 80% of grade 1A and 1B recommendations as well as sepsis bundles suggesting that guideline modification based on available resources may allow implementation of a substantial number of life-saving interventions. However, considering the wide variability of resource availability observed and the regional shortage of essential resources, basic resources need to be supplied to health care facilities to improve sepsis management. Furthermore, establishment of emergency and intensive care departments, adequate staffing and training of health care providers could be further options to improve the care of African patients suffering from severe infection.

## Conclusions

The results of this self-reported survey strongly suggest that the most recent SSC guidelines cannot be implemented in Africa, particularly not in Sub-Sahara Africa, due to a shortage of required hospital facilities, equipment, drugs and disposable materials. However, availability of resources to implement the majority of strong SSC recommendations (grade 1a and 1b) and the sepsis bundles may allow modification of current sepsis guidelines based on available resources and implementation of a substantial number of life-saving interventions into sepsis care in Africa.

## Key messages

• Only a small percentage of African respondents stated that they have the required facilities, equipment, drugs and disposable materials available to implement the Surviving Sepsis Campaign guidelines.

• The Surviving Sepsis Campaign guidelines may not be implementable by African, particularly not by Sub-Saharan African, respondents due to a lack of necessary resources.

• Availability of resources to implement the majority of strong SSC recommendations (grade 1A and 1B) may allow modification of current sepsis guidelines based on available resources and implementation of a substantial number of life-saving interventions into sepsis care in Africa.

## Abbreviations

SSC: Surviving Sepsis Campaign.

## Competing interests

The authors declare that they have no competing interests.

## Authors' contributions

IB designed the study, acquired data, interpreted data, drafted the manuscript and has given final approval of the version to be published. SJ designed the study, acquired data, interpreted data, revised the manuscript for important intellectual content, substantially contributed to revision of the manuscript during the review process and gave final approval of the version to be published. TL made substantial contributions to conception and design of the study, acquired data, interpreted data, revised the manuscript for important intellectual content and gave final approval of the version to be published. DO made substantial contributions to the conception and design of the study, acquired data, interpreted data, revised the manuscript for important intellectual content and gave final approval of the version to be published. JK, IW and TB made substantial contributions to conception and design of the study, acquired data, interpreted data, revised the manuscript for important intellectual content and gave final approval of the version to be published. MWD designed the study, acquired data, analysed data, interpreted data, drafted the manuscript and has given final approval of the version to be published.

## Supplementary Material

Additional file 1 **Electronic supplementary material**. This file contains the study questionnaire; a list of African nations eligible for participation in this survey; hospital facilities, equipment, drugs and disposable materials required to implement single recommendations/suggestions of the Surviving Sepsis Campaign guidelines; and tables on differences between Sub-Saharan African countries and South Africa/Mauritius/Northern African countries. Click here for file
