# Supplementary material for: Availability of critical care resources to treat patients with severe sepsis or septic shock in Africa: a self-reported, continent-wide survey of anaesthesia providers
Source: Crit Care. 2011 Jan 10;15(1):R10. doi: 10.1186/cc9410 (PMC3222039; doi:10.1186/cc9410)
Supplement: Additional file 1 — Electronic supplementary material. This file contains the study questionnaire; a list of African nations eligible for participation in this survey; hospital facilities, equipment, drugs and disposable materials required to implement single recommendations/suggestions of the Surviving Sepsis Campaign guidelines; and tables on differences between Sub-Saharan African countries and South Africa/Mauritius/Northern African countries. [file cc9410-S1.DOC]

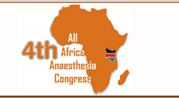


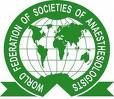


**1. GENERAL INFORMATION**

Name of your hospital? ……………………………………………………………………………..

Country where hospital situated? ……………………………………………………………………………..

Type of hospital? ⭘District ⭘Regional/Provincial ⭘University ⭘Private ⭘Other (please write) ……………..

Number of hospital beds? …………………………………….

What is your grade? ⭘ Physician Anaesthetist ⭘ Other Physician

⭘ Non-Physician Anaesthetist ⭘ Other (please write) ……………………

**2. HOSPITAL FACILITIES**

Does your hospital have the following?

|  | Yes | No | Don’t know |
| --- | --- | --- | --- |
| Emergency / resuscitation room (Room for emergency patient who’s just arrived to hospital) | ⭘ | ⭘ | ⭘ |
| Intensive care unit  (Dedicated unit for critically ill patients) | ⭘ | ⭘ | ⭘ |
| Operating Theatre | ⭘ | ⭘ | ⭘ |

**3. DRUGS**

Are the following drugs available in your hospital?

|  | Always | Sometimes | Never | Don’t know |
| --- | --- | --- | --- | --- |
| IV Ampicillin | ⭘ | ⭘ | ⭘ | ⭘ |
| IV Gentamycin | ⭘ | ⭘ | ⭘ | ⭘ |
| IV Ceftriaxone, Cefotaxime or Ceftazidime | ⭘ | ⭘ | ⭘ | ⭘ |
| IV Piperacillin | ⭘ | ⭘ | ⭘ | ⭘ |
| IV Meropenem or other carbapenem | ⭘ | ⭘ | ⭘ | ⭘ |
| IV Hydrocortisone | ⭘ | ⭘ | ⭘ | ⭘ |
| Sodium Chloride, Ringers Lactate or other crystalloid | ⭘ | ⭘ | ⭘ | ⭘ |
| Gelatine, Dextran or other Colloid | ⭘ | ⭘ | ⭘ | ⭘ |
| Insulin | ⭘ | ⭘ | ⭘ | ⭘ |
|  | Always | Sometimes | Never | Don’t know |
| Oxygen | ⭘ | ⭘ | ⭘ | ⭘ |
| Blood Transfusion | ⭘ | ⭘ | ⭘ | ⭘ |
| Fresh Frozen Plasma | ⭘ | ⭘ | ⭘ | ⭘ |
| Platelets | ⭘ | ⭘ | ⭘ | ⭘ |
| Heparin or Low Molecular Weight Heparin | ⭘ | ⭘ | ⭘ | ⭘ |
| Ranitidine or other H2 receptor blocker | ⭘ | ⭘ | ⭘ | ⭘ |
| Omeprazole or other Proton Pump Inhibitor | ⭘ | ⭘ | ⭘ | ⭘ |
| IV morphine, pethidine or other IV opioid | ⭘ | ⭘ | ⭘ | ⭘ |
| Diazepam | ⭘ | ⭘ | ⭘ | ⭘ |
| Midazolam | ⭘ | ⭘ | ⭘ | ⭘ |
| Propofol | ⭘ | ⭘ | ⭘ | ⭘ |
| Thiopentone | ⭘ | ⭘ | ⭘ | ⭘ |
| Succinylcholine | ⭘ | ⭘ | ⭘ | ⭘ |
| Atracurium or other non-depolarising muscle relaxant | ⭘ | ⭘ | ⭘ | ⭘ |
| Noradrenaline | ⭘ | ⭘ | ⭘ | ⭘ |
| Dopamine | ⭘ | ⭘ | ⭘ | ⭘ |
| Dobutamine | ⭘ | ⭘ | ⭘ | ⭘ |
| Adrenaline | ⭘ | ⭘ | ⭘ | ⭘ |
| Vasopressin | ⭘ | ⭘ | ⭘ | ⭘ |
| Activated Protein C | ⭘ | ⭘ | ⭘ | ⭘ |

**4. PATIENT MONITORING**

Can the following variables be monitored in your hospital?

|  | Always | Sometimes | Never | Don’t know |
| --- | --- | --- | --- | --- |
| Temperature | ⭘ | ⭘ | ⭘ | ⭘ |
| Non-invasive blood pressure | ⭘ | ⭘ | ⭘ | ⭘ |
| Invasive arterial blood pressure | ⭘ | ⭘ | ⭘ | ⭘ |
| Oxygen saturation | ⭘ | ⭘ | ⭘ | ⭘ |
| Central venous pressure | ⭘ | ⭘ | ⭘ | ⭘ |
| Cardiac output | ⭘ | ⭘ | ⭘ | ⭘ |
| Pulmonary arterial pressure | ⭘ | ⭘ | ⭘ | ⭘ |
| End tidal CO_2_ | ⭘ | ⭘ | ⭘ | ⭘ |

**5. LABORATORY**

Can the following investigations be done in your hospital?

|  | Always | Sometimes | Never | Don’t know |
| --- | --- | --- | --- | --- |
| Blood slide for malaria parasites | ⭘ | ⭘ | ⭘ | ⭘ |
| Direct microscopy & gram stain | ⭘ | ⭘ | ⭘ | ⭘ |
| Bacteria culture | ⭘ | ⭘ | ⭘ | ⭘ |
| Antibiotic sensitivities | ⭘ | ⭘ | ⭘ | ⭘ |
| Blood glucose | ⭘ | ⭘ | ⭘ | ⭘ |
| Arterial blood gases | ⭘ | ⭘ | ⭘ | ⭘ |
| Blood lactate | ⭘ | ⭘ | ⭘ | ⭘ |
| Full blood count | ⭘ | ⭘ | ⭘ | ⭘ |
| Creatinine | ⭘ | ⭘ | ⭘ | ⭘ |
| Bilirubin | ⭘ | ⭘ | ⭘ | ⭘ |
| Prothrombin Time (INR) | ⭘ | ⭘ | ⭘ | ⭘ |
| Other coagulation test | ⭘ | ⭘ | ⭘ | ⭘ |

**6. EQUIPMENT**

Is the following equipment available in your hospital?

|  | Always | Sometimes | Never | Don’t know |
| --- | --- | --- | --- | --- |
| X-ray | ⭘ | ⭘ | ⭘ | ⭘ |
| Ultrasound - Abdomen | ⭘ | ⭘ | ⭘ | ⭘ |
| Echocardiography | ⭘ | ⭘ | ⭘ | ⭘ |
| Mechanical Ventilator | ⭘ | ⭘ | ⭘ | ⭘ |
| Syringe pump | ⭘ | ⭘ | ⭘ | ⭘ |
| Fluid infuser/Infusion pump | ⭘ | ⭘ | ⭘ | ⭘ |
| Peritoneal Dialysis | ⭘ | ⭘ | ⭘ | ⭘ |
| Hemodialysis/  hemofiltration | ⭘ | ⭘ | ⭘ | ⭘ |

**7. DISPOSABLES**

Are the following items available in your hospital?

|  | Always | Sometimes | Never | Don’t know |
| --- | --- | --- | --- | --- |
| Venous cannula | ⭘ | ⭘ | ⭘ | ⭘ |
| IV fluid giving set | ⭘ | ⭘ | ⭘ | ⭘ |
| Urinary catheter | ⭘ | ⭘ | ⭘ | ⭘ |
| Gastric tube (NG-tube) | ⭘ | ⭘ | ⭘ | ⭘ |
| Endotracheal tube | ⭘ | ⭘ | ⭘ | ⭘ |
| Oxygen masks | ⭘ | ⭘ | ⭘ | ⭘ |
| Oxygen nasal cannula | ⭘ | ⭘ | ⭘ | ⭘ |
| Central venous catheter | ⭘ | ⭘ | ⭘ | ⭘ |
| Compression stockings | ⭘ | ⭘ | ⭘ | ⭘ |

Thank you very much for participation!


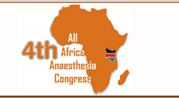


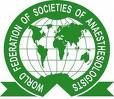


**Table S1.** List of African nations eligible for participation in this survey.

**Eastern Africa:**Burundi, Comoros, Djibouti, Eritrea, Ethiopia, Kenya, Madagascar, Malawi, Mauritius, Mayotte, Mozambique, Réunion, Rwanda, Seychelles, Somalia, Uganda, United Republic of Tanzania, Zambia, Zimbabwe

**Middle Africa:**Angola, Cameroon, Central African Republic, Chad, Congo, Democratic Republic of Congo, Equatorial Guinea, Gabon, Sao Tome and Principe

**Northern Africa:**Algeria, Egypt, Libyan Arab Jamahiriya, Morocco, Sudan, Tunisia, Western Sahara

**Southern Africa:**
Botswana, Lesotho, Namibia, South Africa, Swaziland

**Western Africa:**
Benin, Burkina Faso, Cape Verde, Cote d’Ivoire, Gambia, Ghana, Guinea, Guinea-Bissau, Liberia, Mali, Mauritania, Niger, Nigeria, Saint Helena, Senegal, Sierra Leone, Togo

Source: Reference 32

**Table S2.** Hospital facilities, equipment, drugs and disposable materials required to implement single recommendations/suggestions of the Surviving Sepsis Campaign guidelines.

**Initial resuscitation (first 6 hrs)**

● Begin resuscitation immediately in patients with hypotension or elevated serum lactate 4

mmol/L; do not delay pending intensive care unit admission (1C)

*Materials required: facility to monitor non-invasive or invasive arterial blood pressure and measure lactate levels, intensive care unit*

● Resuscitation goals (1C)

Central venous pressure 8–12 mm Hg

Mean arterial pressure 65 mm Hg

Urine output 0.5 mL*kg-1*hr-1

Central venous (superior vena cava) oxygen saturation 70% or mixed venous 65%

*Materials required: facility to monitor central venous pressure, facility to monitor non-invasive or invasive arterial blood pressure, availability of a urinary catheter, central venous catheter, and facility to measure blood gases*

- If venous oxygen saturation target is not achieved (2C)

Consider further fluid

Transfuse packed red blood cells if required to hematocrit of 30% and/or

Start dobutamine infusion, maximum 20 µg kg-1 min-1

*Materials required: central venous catheter, facility to measure blood gases, availability of IV cannula, IV fluid giving set, crystalloid or colloid solution, blood transfusion and dobutamine*

**Diagnosis**

● Obtain appropriate cultures before starting antibiotics provided this does not significantly

delay antimicrobial administration (1C)

Obtain two or more blood cultures

One or more blood cultures should be percutaneous

One blood culture from each vascular access device in place 48 hrs

Culture other sites as clinically indicated

*Materials required: direct microscopy and gram stain, bacteria culture*

● Perform imaging studies promptly to confirm and sample any source of infection, if safe to do so (1C)

*Materials required: availability of x-ray and ultrasound*

**Antibiotic therapy**

● Begin intravenous antibiotics as early as possible and always within the first hour of

recognizing severe sepsis (1D) and septic shock (1B)

*Materials required: availability of at least one antibiotic drug*

● Broad-spectrum: one or more agents active against likely bacterial/fungal pathogens and with good penetration into presumed source (1B)

*Materials required: availability of either ceftriaxone/cefotaxime/ceftazidime, piperacilline or meropenem/other carbapenem*

● Reassess antimicrobial regimen daily to optimize efficacy, prevent resistance, avoid toxicity, and minimize costs (1C)
*Materials required: none*

- Consider combination therapy in *Pseudomonas* infections (2D)

*Materials required: bacteria culture and availability of at least two of the following antibiotic drugs: ceftriaxone/cefotaxime/ceftazidime, piperacilline, meropenem/other carbapenem, or gentamycin*

- Consider combination empiric therapy in neutropenic patients (2D)

*Materials required: facility to measure full blood count and at least two antibiotic drugs*

- Combination therapy 3–5 days and de-escalation following susceptibilities (2D)

*Materials required: facility to determine antibiotic sensitivities*

● Duration of therapy typically limited to 7–10 days; longer if response is slow or there are

undrainable foci of infection or immunologic deficiencies (1D)
*Materials required: none*

● Stop antimicrobial therapy if cause is found to be noninfectious (1D)
*Materials required: facility to perform bacterial cultures*

**Source identification and control**

● A specific anatomic site of infection should be established as rapidly as possible (1C) and

within first 6 hrs of presentation (1D)

*Materials required: availability of x-ray and ultrasound*

● Formally evaluate patient for a focus of infection amenable to source control measures (e.g.

abscess drainage, tissue debridement) (1C)

*Materials required: availability of x-ray and ultrasound*

● Implement source control measures as soon as possible following successful initial

resuscitation (1C) (exception: infected pancreatic necrosis, where surgical intervention is best

delayed) (2B)

*Materials required: operation room*

● Choose source control measure with maximum efficacy and minimal physiologic upset (1D)

*Materials required: none*

● Remove intravascular access devices if potentially infected (1C)

*Materials required: none*

**Fluid therapy**

● Fluid-resuscitate using crystalloids or colloids (1B)

*Materials required: availability of IV cannula, IV fluid giving set, crystalloid or colloid solution*

● Target a central venous pressure of 8 mm Hg (12 mm Hg if mechanically ventilated) (1C)
*Materials required: facility to measure central venous pressure*

● Use a fluid challenge technique while associated with a hemodynamic improvement (1D)
*Materials required: availability of IV cannula, IV fluid giving set, crystalloid or colloid solution and facility to monitor non-invasive or invasive arterial blood pressure*

● Give fluid challenges of 1000 mL of crystalloids or 300–500 mL of colloids over 30 mins. More rapid and larger volumes may be required in sepsis-induced tissue hypoperfusion (1D)

*Materials required: availability of IV cannula, IV fluid giving set, crystalloid or colloid solution*

● Rate of fluid administration should be reduced if cardiac filling pressures increase without

concurrent hemodynamic improvement (1D)

*Materials required: availability of IV cannula, IV fluid giving set, crystalloid or colloid solution, facility to measure central venous pressure*

**Vasopressors**

● Maintain mean arterial pressure 65 mm Hg (1C)

*Materials required: facility to measure non-invasive or invasive arterial blood pressure*

● Norepinephrine and dopamine centrally administered are the initial vasopressors of choice (1C)

*Materials required: availability of a central venous catheter, norepinephrine or dopamine*

- Epinephrine, phenylephrine, or vasopressin should not be administered as the initial

vasopressor in septic shock (2C). Vasopressin 0.03 units/min may be subsequently added to

norepinephrine with anticipation of an effect equivalent to norepinephrine alone
*Materials required: availability of vasopressin*

- Use epinephrine as the first alternative agent in septic shock when blood pressure is

poorly responsive to norepinephrine or dopamine (2B).
*Materials required: facility to monitor non-invasive or invasive arterial blood pressure, availability of epinephrine*

● Do not use low-dose dopamine for renal protection (1A)
*Materials required: none*

● In patients requiring vasopressors, insert an arterial catheter as soon as practical (1D)
*Materials required: facility to monitor invasive arterial blood pressure*

**Inotropic therapy**

● Use dobutamine in patients with myocardial dysfunction as supported by elevated cardiac

filling pressures and low cardiac output (1C)
*Materials required: facility to monitor central venous pressure or cardiac output, availability of dobutamine*

● Do not increase cardiac index to predetermined supranormal levels (1B)

*Materials required: none*

**Corticosteroids**

- Consider intravenous hydrocortisone for adult septic shock when hypotension responds

poorly to adequate fluid resuscitation and vasopressors (2C)

*Materials required: facility to measure non-invasive or invasive arterial blood pressure, availability of IV cannula, IV fluid giving set, availability of crystalloid or colloid solution, availability of a central venous catheter, dopamine or norepinephrine, hydrocortisone*

- ACTH stimulation test is not recommended to identify the subset of adults with septic

Shock who should receive hydrocortisone (2B)
*Materials required: none*

- Hydrocortisone is preferred to dexamethasone (2B)

*Materials required: availability of hydrocortisone*

- Fludrocortisone (50 g orally once a day) may be included if an alternative to

hydrocortisone is being used that lacks significant mineralocorticoid activity. Fludrocortisone is optional if hydrocortisone is used (2C)
*Materials required: none*

- Steroid therapy may be weaned once vasopressors are no longer required (2D)

*Materials required: availability of hydrocortisone, availability of a central venous catheter, dopamine or norepinephrine*

● Hydrocortisone dose should be 300 mg/day (1A)

*Materials required: availability of hydrocortisone*

● Do not use corticosteroids to treat sepsis in the absence of shock unless the patient’s

endocrine or corticosteroid history warrants it (1D)
*Materials required: none*

**Recombinant human activated protein C (rhAPC)**

- Consider rhAPC in adult patients with sepsis-induced organ dysfunction with clinical

assessment of high risk of death (typically APACHE II 25 or multiple organ failure) if there

are no contraindications (2B, 2C for postoperative patients).

*Materials required: facility to measure body temperature, non-invasive or invasive arterial blood pressure, arterial blood gases, creatinine, and full blood count, availability of recombinant human activated protein C*

● Adult patients with severe sepsis and low risk of death (typically, APACHE II 20 or one

organ failure) should not receive rhAPC (1A)

*Materials required: facility to measure body temperature, non-invasive or invasive arterial blood pressure, arterial blood gases, creatinine, and full blood count*

**Blood product administration**

● Give red blood cells when hemoglobin decreases to 7.0 g/dL (70 g/L) to target a hemoglobin of 7.0–9.0 g/dL in adults (1B). A higher hemoglobin level may be required in special circumstances (e.g., myocardial ischaemia, severe hypoxemia, acute hemorrhage, cyanotic heart disease, or lactic acidosis)

*Materials required: facility to measure full blood count, availability of IV cannula, IV fluid giving set and blood transfusion*

- Do not use erythropoietin to treat sepsis-related anemia. Erythropoietin may be used for

other accepted reasons (1B)

*Materials required: none*

- Do not use fresh frozen plasma to correct laboratory clotting abnormalities unless there is

bleeding or planned invasive procedures (2D)
*Materials required: availability of IV cannula, IV fluid giving set and fresh frozen plasma*

● Do not use antithrombin therapy (1B)

*Materials required: none*

- Administer platelets when (2D)

Counts are 5000/mm3 (5 x 10^9^/L) regardless of bleeding

Counts are 5000–30,000/mm3 (5–30 x 10^9^/L) and there is significant bleeding risk

Higher platelet counts (50,000/mm3 [50 x 10^9^/L]) are required for surgery or invasive procedures

*Materials required: availability of IV cannula, IV fluid giving set and platelets*

**Mechanical ventilation of sepsis-induced ALI/ARDS**

● Target a tidal volume of 6 mL/kg (predicted) body weight in patients with ALI/ARDS (1B)

*Materials required: facility to measure oxygen saturation or arterial blood gases, availability of endotracheal tube and mechanical ventilator*

● Target an initial upper limit plateau pressure 30 cmH2O. Consider chest wall compliance when assessing plateau pressure (1C)

*Materials required: availability of endotracheal tube and mechanical ventilator*

● Allow PaCO2 to increase above normal, if needed, to minimize plateau pressures and tidal volumes (1C)

*Materials required: facility to measure arterial blood gases or endtidal CO_2_, availability of endotracheal tube and mechanical ventilator*

● Set PEEP to avoid extensive lung collapse at end-expiration (1C)

*Materials required: availability of endotracheal tube and mechanical ventilator*

- Consider using the prone position for ARDS patients requiring potentially injurious levels

of FIO2 or plateau pressure, provided they are not put at risk from positional changes (2C)

*Materials required: availability of endotracheal tube and mechanical ventilator*

● Maintain mechanically ventilated patients in a semirecumbent position (head of the bed raised to 45°) unless contraindicated (1B), between 30° and 45° (2C)
*Materials required: none*

- Noninvasive ventilation may be considered in the minority of ALI/ARDS patients with

mild to moderate hypoxemic respiratory failure. The patients need to be hemodynamically stable, comfortable, easily arousable, able to protect/clear their airway, and expected to recover rapidly (2B)

*Materials required: facility to monitor oxygen saturation or arterial blood gases and non-invasive or invasive arterial blood pressure, availability of mechanical ventilator*

● Use a weaning protocol and an SBT regularly to evaluate the potential for discontinuing mechanical ventilation (1A)

*Materials required: availability of endotracheal tube and mechanical ventilator*

● Spontaneous breathing trial options include a low level of pressure support with continuous positive airway pressure 5 cm H2O or a T piece

*Materials required: availability of endotracheal tube and mechanical ventilator*

● Before the spontaneous breathing trial, patients should

be arousable

be hemodynamically stable without vasopressors

have no new potentially serious conditions

have low ventilatory and end-expiratory pressure requirement

require FiO2 levels that can be safely delivered with a face mask or nasal cannula

*Materials required: facility to monitor non-invasive or invasive arterial blood pressure, availability of endotracheal tube and mechanical ventilator, oxygen, oxygen mask or nasal cannula*

● Do not use a pulmonary artery catheter for the routine monitoring of patients with ALI/ARDS (1A)

*Materials required: none*

● Use a conservative fluid strategy for patients with established ALI who do not have evidence of tissue hypoperfusion (1C)

*Materials required: availability of IV cannula, IV fluid giving set, crystalloid or colloid solution*

**Sedation, analgesia, and neuromuscular blockade in sepsis**

● Use sedation protocols with a sedation goal for critically ill mechanically ventilated patients (1B)

*Materials required: none*

● Use either intermittent bolus sedation or continuous infusion sedation to predetermined end points (sedation scales), with daily interruption/lightening to produce awakening. Re-titrate if necessary (1B)

*Materials required: availability of IV morphine/opioid, benzodiazepine, propofol or thiopentone*

● Avoid neuromuscular blockers where possible. Monitor depth of block with train-of-four when using continuous infusions (1B)

*Materials required: availability of non-depolarizing muscle relaxant*

**Glucose control**

● Use intravenous insulin to control hyperglycemia in patients with severe sepsis following stabilization in the ICU (1B)

*Materials required: facility to measure blood glucose, availability of insulin*

● Aim to keep blood glucose 150 mg/dL (8.3 mmol/L) using a validated protocol for insulin dose adjustment (2C)

*Materials required: facility to measure blood glucose, availability of insulin*

● Provide a glucose calorie source and monitor blood glucose values every 1–2 hrs (4 hrs when stable) in patients receiving intravenous insulin (1C)

*Materials required:* *facility to measure blood glucose, availability of insulin*

● Interpret with caution low glucose levels obtained with point of care testing, as these techniques may overestimate arterial blood or plasma glucose values (1B)

*Materials required: facility to measure blood glucose*

**Renal replacement**

- Intermittent hemodialysis and continuous veno-venous hemofiltration are considered equivalent (2B)

*Materials required: availability of hemodialysis or hemofiltration*

- Continuous veno-venous hemofiltration offers easier management in hemodynamically unstable patients (2D)

*Materials required: facility to measure non-invasive or invasive arterial blood pressure, availability of hemodialysis or hemofiltration*

**Bicarbonate therapy**

● Do not use bicarbonate therapy for the purpose of improving hemodynamics or reducing vasopressor requirements when treating hypoperfusion-induced lactic acidemia with pH 7.15 (1B)

*Materials required: none*

**Deep vein thrombosis prophylaxis**

● Use either low-dose unfractionated heparin or low molecular weight heparin, unless contraindicated (1A)

*Materials required: availability of unfractionated or low molecular weight heparin*

● Use a mechanical prophylactic device, such as compression stockings or an intermittent compression device, when heparin is contraindicated (1A)

*Materials required: availability of compression stockings*

- Use a combination of pharmacologic and mechanical therapy for patients who are at very

high risk for deep vein thrombosis (2C)

*Materials required: availability of unfractionated or low molecular weight heparin and compression stockings*

- In patients at very high risk, low molecular weight heparin should be used rather than unfractionated heparin (2C)

*Materials required: availability of low molecular weight heparin*

**Stress ulcer prophylaxis**

● Provide stress ulcer prophylaxis using H2 blocker (1A) or proton pump inhibitor (1B). Benefits of prevention of upper gastrointestinal bleed must be weighed against the potential for development of ventilator-acquired pneumonia

*Materials required: availability of ranitidine/other H2 blocker or omeprazole/other proton pump inhibitor*

**Consideration for limitation of support**

● Discuss advance care planning with patients and families. Describe likely outcomes and set realistic expectations (1D)

*Materials required: none*

Numbers and letters in parentheses denote the strength of the recommendation. The Surviving Sepsis Campaign categorized the strength of their recommendations using the GRADE system which classifies recommendations as strong (grade 1) or weak (grade 2). Furthermore, the system Classifies the quality of evidence as high (grade A), moderate (grade B), low (grade C), or very low (grade D).The grade of strong or weak is considered of greater clinical importance than a difference in letter level of quality of evidence.

**SEVERE SEPSIS BUNDLES**

**Sepsis Resuscitation Bundle**

**Bundle Element “Lactate”** Measure serum lactate

*Materials required: facility to measure lactate levels*

**Bundle Element “Cultures”** Obtain blood cultures prior to antibiotic administration

*Materials required:* *facility to perform bacterial cultures, availability of at least one antibiotic drug*

**Bundle Element “Antibiotics”** Administer broad-spectrum antibiotic within 3 hours of ED admission and within 1 hour of non-ED admission

*Materials required: availability of either ceftriaxone/cefotaxime/ceftazidime, piperacilline or meropenem/other carbapenem*

**Bundle Element “Hypotension”** Treat hypotension and/or elevated lactate with fluids

*Materials required: facility to monitor non-invasive or invasive arterial blood pressure or measure lactate levels, availability of IV cannula, IV fluid giving set, crystalloid or colloid solution*

**Bundle Element “CVP/ScvO2”** Maintain adequate central venous pressure and central venous oxygen saturation

*Materials required: central venous catheter, facility to monitor central venous pressure, facility to measure blood gases*

**Sepsis Management Bundle**

**Bundle Element “Steroids”** Administer low-dose steroids for septic shock in accordance with a standardized ICU policy

*Materials required: availability of hydrocortisone*

**Bundle Element “rhAPC”** Administer recombinant human activated protein C (rhAPC) in accordance with a standardized ICU policy

*Materials required: availability of recombinant human activated protein C*

**Bundle Element “Glucose”** Maintain glucose control lower limit of normal, but <180 mg/dL (10 mmol/L)

*Materials required: facility to measure blood glucose, availability of insulin*

**Bundle Element “Plateau Pressure”** Maintain a median inspiratory plateau pressure (IPP) <30 cmH2O for mechanically ventilated patients

*Materials required: availability of endotracheal tube and mechanical ventilator*
